# Curcumin-, Anthocyanin-, and Carotenoid-Based Active and Intelligent Packaging Systems for Food Preservation and Freshness Monitoring

**DOI:** 10.3390/foods15152630

**Published:** 2026-07-27

**Authors:** Xiaocheng Pan, Yangkun Xing, Jianfeng Wang, Yuhan Luo, Dongting Ma, Haotian Wu, Bo Xia

**Affiliations:** 1 Jiyang College, Zhejiang A&F University, Zhuji 311800, China; 2 Xingzhi College, Zhejiang Normal University, Lanxi 321100, China; 3 School of Food Science and Technology, Jiangnan University, Wuxi 214122, China

**Keywords:** colorimetric indicators, pH-responsive films, antioxidant and antimicrobial activity, shelf-life extension

## Abstract

Increasing consumer demand for safer and more sustainable food preservation has stimulated interest in natural pigments as multifunctional components in active and intelligent food systems. This review critically summarizes curcumin-, anthocyanin-, and carotenoid-based systems for food preservation and freshness monitoring, focusing on their mechanisms, material strategies, food-matrix-dependent performance, and technological challenges. Curcumin mainly contributes antioxidant, antimicrobial, and photoresponsive functions, especially photodynamic inactivation when supported by suitable carriers. Anthocyanins provide pH- and amine-responsive colorimetric signals for intelligent freshness monitoring, whereas carotenoids primarily protect oxidation-sensitive foods through radical quenching and lipid oxidation inhibition. Recent incorporation of these pigments into biopolymer films, edible coatings, emulsions, nanocarriers, hydrogels, aerogels, cyclodextrin complexes, metal–organic frameworks, and deep eutectic solvent-assisted systems has improved pigment stability, dispersion, barrier performance, and controlled release. However, poor solubility, environmental instability, matrix interference, migration safety, sensory impacts, quantitative calibration, and scalability remain unresolved. Future development should emphasize mechanism-guided formulation, real-food validation, and safe, stable, scalable pigment-based systems tailored to specific deterioration pathways and commercial applications.

## 1. Introduction

Growing consumer concern about synthetic food additives, conventional chemical preservatives, and petroleum-derived packaging materials has accelerated the search for safer, more environmentally compatible, and more sustainable alternatives in food systems. In this context, natural compounds, including polysaccharides, peptides, polyphenols, essential oils, and natural pigments, have attracted broad interest owing to their renewable origins, perceived safety, biodegradability, and diverse functional properties [1,2,3]. Among these compounds, natural pigments are particularly attractive because they can act not only as color-imparting substances but also as functional agents with antioxidant, antimicrobial, photoresponsive, or freshness-indicating properties. These multifunctional characteristics make them promising candidates for active preservation, intelligent quality monitoring, and sustainable food packaging applications [3,4,5]. Nevertheless, the practical use of natural pigments in complex food systems remains challenging. Many pigments show poor aqueous solubility, sensitivity to light, oxygen, heat, or pH, limited compatibility with hydrophilic matrices, and uncontrolled migration or release. To overcome these limitations, recent studies have incorporated natural pigments into biopolymer films, edible coatings, nanocarriers, Pickering emulsions, hydrogels, aerogels, electrospun fibers, cyclodextrin complexes, metal–organic frameworks, and deep eutectic solvent-assisted systems, thereby improving pigment dispersion, stability, controlled release, barrier performance, and food-related functionality [1,3,6,7,8,9,10,11]. These advances suggest that the performance of natural pigments is determined not only by their intrinsic bioactivity, but also by the compatibility between pigment chemistry, material platform, and food matrix. This relationship provides the basis for understanding why different natural pigments require distinct formulation strategies and fulfill different functions in food applications.

**Figure 1:**
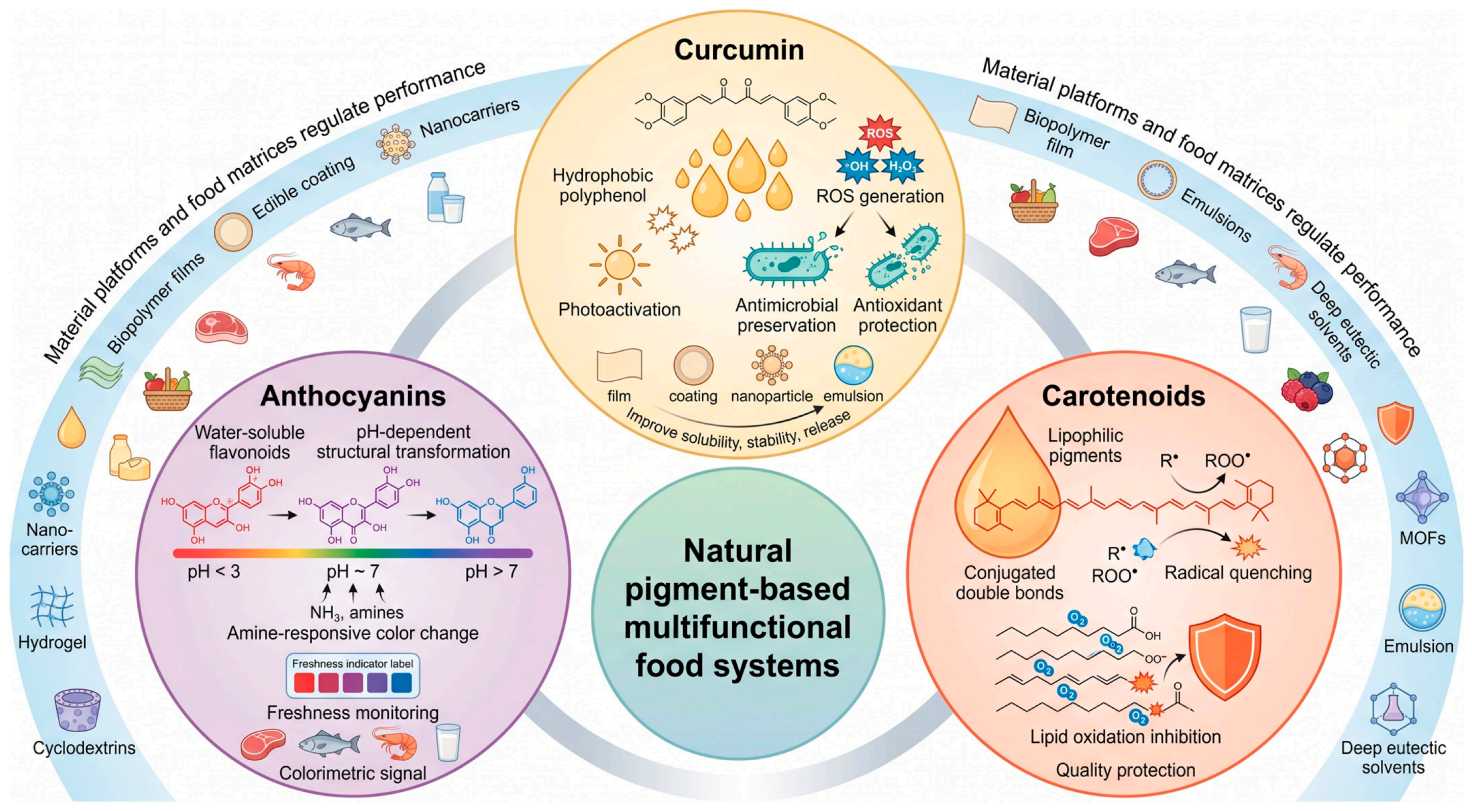
Comparative functional mechanisms of curcumin, anthocyanins, and carotenoids in food preservation and freshness monitoring.

Among the natural pigments investigated for food applications, curcumin, anthocyanins, and carotenoids have received particular attention because they represent different physicochemical properties, structural features, and functional mechanisms (Figure 1). Curcumin is a hydrophobic diarylheptanoid polyphenol derived from Curcuma longa, and its conjugated structure and phenolic groups contribute to antioxidant, antimicrobial, and light-activated photosensitizing functions. For instance, curcumin-mediated photodynamic inactivation inhibited Listeria monocytogenes on salmon and delayed pH increase, total volatile basic nitrogen formation, lipid hydrolysis, protein degradation, color change, and water loss during storage [4]. Curcumin has also been incorporated into cinnamaldehyde-loaded Pickering emulsions and photodynamic systems for microbial control in peanuts and fresh Tremella fuciformis, demonstrating its potential in non-thermal preservation and active food systems [1,12]. In contrast, anthocyanins are water-soluble flavonoid pigments whose pH-dependent structural transformations generate visible color changes, giving them a clear advantage in intelligent packaging and freshness monitoring. Anthocyanin-containing films and biofilms have been applied to beef, strawberries, bananas, and shrimp, where they combine pH- or amine-responsive indication with antioxidant, antibacterial, or shelf-life-extending effects [2,5,13]. Carotenoids, including astaxanthin, lycopene, and lutein, are lipophilic pigments characterized by extended conjugated double-bond systems, which make them effective antioxidants for reducing oxidative deterioration. Astaxanthin-based coatings and films have been used for strawberry and shrimp preservation [3,6], while lycopene microcapsule-loaded films have been applied to blueberries and sweet cherries to improve the usability of this hydrophobic and oxidation-sensitive pigment [14,15]. Therefore, these pigment groups should not be regarded simply as alternative natural colorants. Rather, they represent distinct functional pathways in food applications: curcumin is mainly associated with photoresponsive antimicrobial preservation, anthocyanins with freshness-responsive colorimetric monitoring, and carotenoids with antioxidant-oriented quality protection.

Despite these advances, the current literature on natural pigment-based food systems remains scattered across different pigment types, material platforms, and food categories. Many studies evaluate one pigment or one packaging format in a specific food matrix, which makes it difficult to compare how different pigment classes respond to diverse deterioration pathways such as respiration-related senescence in fruits, oxidative and microbial spoilage in meats, volatile nitrogen accumulation in seafood, or acidification in dairy products. In addition, the functional effects observed in composite systems are not always attributable to the pigment alone. Polymers, essential oils, polyphenols, antimicrobial peptides, nanoparticles, cyclodextrins, metal–organic frameworks, deep eutectic solvents, and multilayer structures may also play important roles in antimicrobial activity, antioxidant protection, barrier enhancement, mechanical reinforcement, controlled release, or color stabilization [5,6]. Therefore, a comparative and mechanism-oriented review is needed to clarify how pigment chemistry, material design, and food-matrix characteristics jointly determine preservation or monitoring performance. This review systematically compares curcumin-, anthocyanin-, and carotenoid-based systems from the perspectives of mechanisms, material strategies, food-matrix-dependent applications, and technological challenges, aiming to guide the development of safe, stable, scalable, and application-oriented pigment-based food systems. By connecting the dominant functions of each pigment group with the deterioration characteristics of fruits, meats, seafood, dairy products, and other foods, this review aims to distinguish pigment-derived contributions from carrier- or matrix-derived effects and to provide guidance for the rational development of safe, stable, scalable, and application-oriented natural pigment-based food systems.

## 2. Curcumin-Based Active Applications

**Figure 2:**
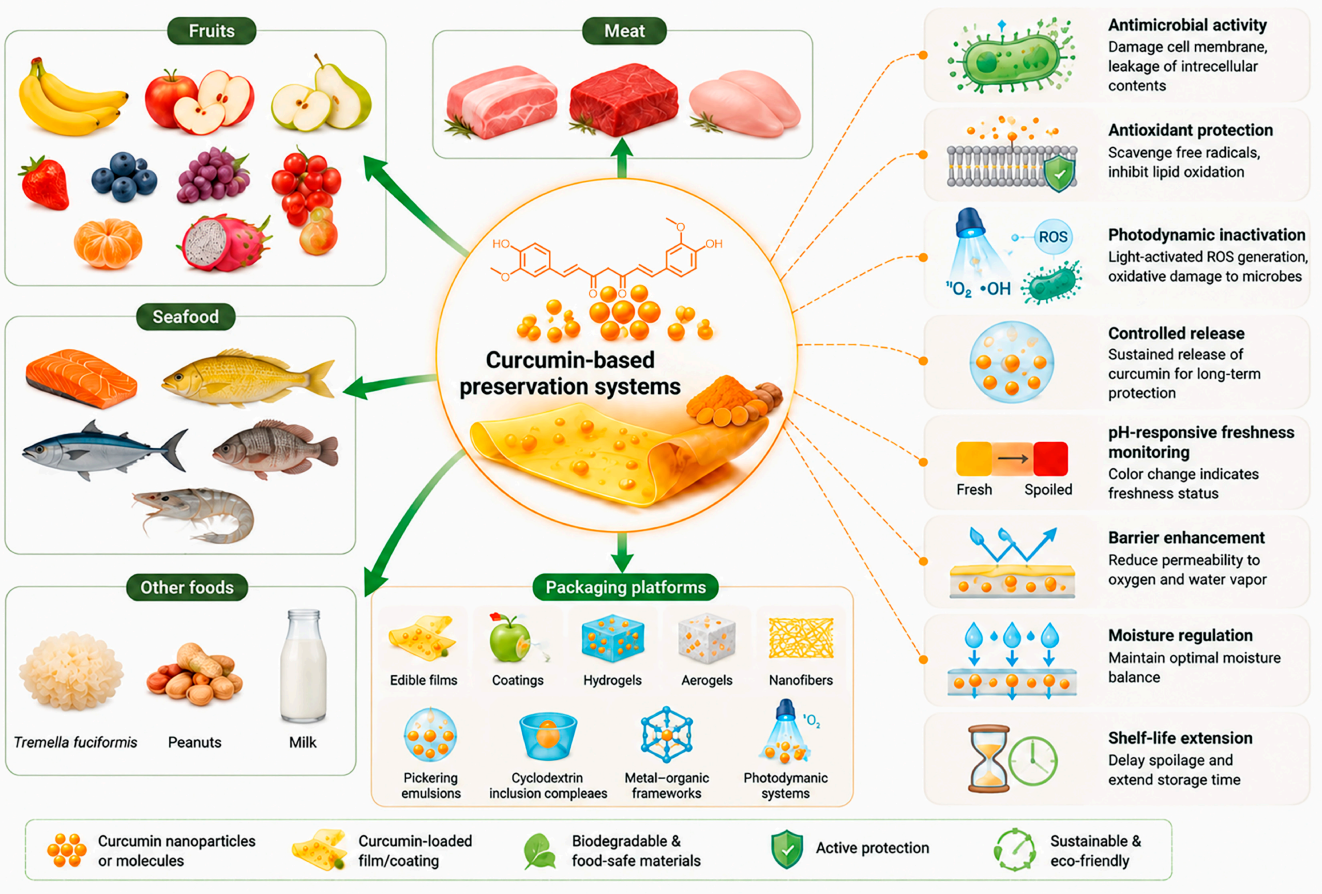
Schematic overview of curcumin-based preservation systems in different food matrices: carrier-assisted stabilization, controlled release, photodynamic inactivation, antioxidant protection, antimicrobial activity, and freshness-related color responses.

Curcumin, a yellow diarylheptanoid polyphenol derived from the rhizomes of Curcuma longa, has emerged as a versatile natural pigment for food preservation because of its antioxidant, antimicrobial, and light-responsive properties. In food matrices, curcumin can attenuate lipid and protein oxidation, restrict spoilage microorganisms, and, under suitable formulation conditions, provide photoactivated antimicrobial effects or pH-related colorimetric responses. These characteristics make it suitable for active packaging and, in selected cases, intelligent monitoring systems (Figure 2). Its direct application, however, is limited by poor aqueous solubility, modest chemical stability, and susceptibility to degradation under light, oxygen, alkaline pH, heat, and complex food-matrix conditions. Therefore, most curcumin-based systems depend on delivery architectures such as films, coatings, carriers, hydrogels, aerogels, emulsions, and inclusion complexes. These platforms enhance dispersion, protect curcumin from degradation, regulate release or activation, and determine whether its antioxidant, antimicrobial, photodynamic, or sensing functions can be expressed at the food interface.

### 2.1. Fruits

#### 2.1.1. Curcumin-Based Coatings for Climacteric Fruits

In climacteric fruits, curcumin-based preservation should be interpreted in relation to respiration-driven ripening, moisture loss, microbial contamination, and surface-quality deterioration rather than as a uniform antioxidant or antimicrobial intervention. Current curcumin-based strategies for banana preservation mainly rely on chitosan-derived films or coatings in which curcumin is combined with nanocarriers or inorganic fillers rather than used as a free additive [16,17]. In these systems, chitosan provides the edible structural matrix and basic antimicrobial protection, while curcumin-loaded or curcumin-modified nanostructures improve stability, release behavior, antibacterial activity, and barrier performance. Thus, the main conclusion from banana studies is not that curcumin alone is sufficient for climacteric-fruit preservation, but that curcumin requires a compatible matrix and stabilizing carriers to function effectively at the fruit surface. Current evidence, however, remains focused mainly on mass loss, appearance, storage duration, and antibacterial activity, while the influence of these materials on ripening-related physiological changes still requires clarification. As a result, the reported delay in banana deterioration should be interpreted cautiously, because it may arise from reduced moisture loss and microbial pressure rather than from direct control of climacteric ripening.

Fresh-cut climacteric fruits present a different preservation context because cutting disrupts tissue integrity and increases susceptibility to water loss, microbial contamination, and respiration-related quality changes. For apple slices, curcumin-loaded porous starch in a chitosan matrix was used to regulate gas transfer and create a low-O_2_/high-CO_2_ microenvironment [18]. In fresh-cut pears, by contrast, curcumin served mainly as a photosensitizer for photodynamic inactivation of L. monocytogenes 19]. These examples indicate that carrier-assisted curcumin can be adapted to different preservation needs in fresh-cut climacteric fruits. However, the dominant mechanism is not always easy to assign. In apple slices, the benefit may be closely linked to matrix-controlled gas regulation, whereas in fresh-cut pears it is more directly related to photodynamic antimicrobial activity. Future comparisons should therefore distinguish more clearly among pigment-derived activity, carrier-mediated effects, and microenvironment regulation.

#### 2.1.2. Curcumin-Based Systems for Non-Climacteric Fruits

For non-climacteric fruits, curcumin-based packaging is less related to ripening control and more closely associated with water loss, softening, surface decay, oxidative deterioration, and maintenance of visual quality. Strawberry has often been used as a model because its fragile tissue and high moisture content make it highly susceptible to microbial spoilage and texture loss. In strawberry applications, curcumin is generally more effective when incorporated into structured delivery formats rather than directly blended into hydrophilic films. Nanoparticle, MOF-based, and cyclodextrin-assisted systems have been used to improve curcumin dispersion, pH-responsive release, UV shielding, barrier properties, and antibacterial activity [17,18,19]. Bacterial cellulose-based films, electrospun nanofibers, and Fe-DNA nanonetwork-assisted coatings have also been developed to improve curcumin compatibility, water dispersibility, antioxidant activity, and antimicrobial performance [20,21,22]. Although these systems differ in composition, they are all used to improve the compatibility and stability of curcumin in aqueous or hydrophilic packaging matrices. Thus, strawberry studies support a carrier-enabled role of curcumin, in which preservation is closely linked to improved pigment stability, dispersion, release behavior, barrier properties, and antimicrobial performance.

In blueberries, curcumin-based films appear to act through both surface protection and possible regulation of oxidative status. Curcumin-loaded starch films and Pickering emulsion-based alginate films improved barrier, antioxidant, antimicrobial, and quality-retention properties [23,24]. One study further suggested that curcumin-loaded films may delay blueberry senescence by supporting endogenous antioxidant defenses, including antioxidant enzyme activity and the AsA-GSH cycle [23]. Zein/chitosan films containing curcumin and eugenol combining UV blocking, microbial inhibition, firmness retention, and pH-responsive color change [25]. Compared with the strawberry literature, the blueberry studies provide somewhat stronger evidence for a possible link between curcumin delivery and endogenous antioxidant regulation. However, this interpretation remains tentative because barrier control, UV shielding, moisture regulation, and coactive compounds such as eugenol may also contribute to quality retention.

In grapes, surface decay and microbial contamination are usually more relevant targets than ripening modulation. For varieties such as Kyoho grapes, thin skin, juicy texture, and high sugar content make microbial spoilage a major challenge. Curcumin inclusion complexes and electrospun fibrous membranes have been used to improve water compatibility, environmental stability, oxygen barrier capacity, long-lasting antioxidant activity, antibacterial performance, and UV protection [26,27]. In contrast to climacteric-fruit applications, where gas regulation and ripening-related deterioration are more relevant, grape preservation depends more on maintaining stable surface activity against microbial decay. Beyond berries and grapes, curcumin-based approaches have been extended to other perishable produce with distinct storage microenvironments, including cherry tomatoes, tangerines, and dragon fruit. pH-sensitive, thermally controlled, and moisture-responsive curcumin systems have been used to improve loading, release, barrier properties, and antibacterial effects [28,29,30]. These studies also suggest that curcumin packaging is moving beyond simple incorporation of the pigment. Increasingly, pH, temperature, and moisture changes are used to trigger release and to reflect the local spoilage environment. However, these responsive designs also make the mechanism more difficult to separate, because release behavior, carrier degradation, antimicrobial action, and barrier changes may occur at the same time. Future comparisons should therefore include free curcumin, unloaded carriers, carrier-loaded curcumin, and complete composite systems within the same fruit matrix.

### 2.2. Meats

Compared with fruits, where respiration and moisture regulation are major concerns, fresh meat deterioration is driven mainly by microbial proliferation, lipid and protein oxidation, myoglobin oxidation, drip loss, discoloration, and spoilage-related metabolite accumulation during refrigerated storage. These processes are closely connected: microbial growth accelerates pH changes and off-odor formation, oxidative reactions impair flavor, texture, and nutritional value, and pigment oxidation directly affects visual acceptability. Curcumin is relevant in this context because it can scavenge free radicals, inhibit spoilage microorganisms, participate in light-activated photodynamic inactivation, and respond to pH changes associated with meat freshness. Its poor aqueous solubility, environmental instability, and weak compatibility with hydrophilic packaging matrices, however, limit direct use. Recent studies have therefore embedded curcumin in films, hydrogels, aerogels, emulsions, inclusion complexes, metal-chelated structures, and nanoencapsulation platforms. These systems improve curcumin dispersion and stability, regulate its migration or release at the meat surface, and allow antioxidant, antimicrobial, photodynamic, and freshness-indicating functions to operate in a coordinated manner. Curcumin-based meat preservation is therefore better understood as the construction of multifunctional food-packaging interfaces rather than the simple addition of a natural pigment.

#### 2.2.1. Curcumin-Based Systems for Pork Preservation

In pork preservation, curcumin-based formulations have mainly been developed to control microbial growth, oxidative deterioration, exudate accumulation, texture loss, and freshness-related pH changes. One major strategy is to construct multifunctional films in which curcumin contributes antioxidant and antibacterial activity as well as pH-responsive color variation. Polymer-assisted dispersion, complexation, and metal-chelation strategies have been used to improve curcumin compatibility, reduce aggregation, enhance thermal or interfacial stability, and retain its response to spoilage-associated alkaline changes [31,32]. During pork storage, these systems delayed increases in TVB-N (total volatile basic nitrogen formation) and pH, reduced texture deterioration, and provided visible color changes. These studies indicate that the preservation and sensing effects arise from the combined action of curcumin chemistry and matrix-mediated stabilization rather than from free curcumin alone.

Another direction is to position curcumin within reinforced biopolymer networks, adhesive coatings, or light-responsive carriers to maintain its activity at the meat surface. Pickering emulsion-containing films, adhesive coatings, carbon dot-assisted hydrogels, hydrophobic cellulose films, and ferric–curcumin nanocomposites have been used to improve water resistance, surface retention, photostability, singlet oxygen generation, or photothermal antibacterial activity [33,34,35,36,37]. These examples show that curcumin can be expanded from a passive antioxidant or pH-responsive pigment to an activatable antimicrobial agent. However, curcumin-mediated pork preservation depends strongly on carrier design, surface adhesion, photostability, irradiation conditions, and the accessibility of curcumin to microorganisms on moist meat surfaces.

Beyond conventional films and coatings, absorbent biodegradable formats are relevant to pork because exudate accumulation promotes microbial proliferation, off-odor formation, and quality loss in tray packaging. Curcumin-containing aerogels and cyclodextrin-assisted films have been developed to combine exudate uptake, sustained antibacterial activity, photodynamic function, and biodegradability [38,39]. Taken together, pork-related studies indicate a shift from simple active-agent incorporation toward multifunctional interfaces that integrate oxidation control, microbial inhibition, exudate management, light activation, biodegradability, and freshness indication. Remaining challenges include maintaining curcumin activity under retail conditions, regulating release or activation at the meat surface, and confirming whether laboratory-scale improvements can translate into robust commercial preservation.

#### 2.2.2. Curcumin-Mediated Preservation of Beef

For beef preservation, curcumin-based strategies mainly target psychrotrophic spoilage bacteria, oxidative and proteolytic deterioration, discoloration, and freshness-related pH changes. Compared with pork studies, where exudate management and tray-packaging formats are frequently emphasized, beef applications place greater attention on microbial community shifts, brown color development, and spoilage-metabolite control during chilled storage. Curcumin-mediated photodynamic inactivation has been enhanced by mild acidification or nanoencapsulation, which improves curcumin availability, microbial contact, controlled release, and blue-light-responsive ROS (reactive oxygen species) generation [40,41]. These studies suggest that curcumin-based beef preservation can be effective when photosensitization is supported by formulation strategies that increase curcumin stability and interaction with spoilage microorganisms.

Film-based formats further show how curcumin can integrate active preservation with freshness-related visual response in beef packaging. Curcumin-containing biopolymer and bilayer films have improved mechanical strength, barrier performance, antioxidant capacity, antibacterial activity, slow release, UV shielding, and pH-sensitive color changes [42,43]. These examples indicate that curcumin can act simultaneously as an active compound, structural modifier, controlled-release component, and pH-responsive freshness marker in beef packaging. Further work is still needed to clarify how curcumin-based PDI (photodynamic inactivation) or colorimetric systems perform under variable retail illumination, different packaging atmospheres, and beef cuts with heterogeneous surfaces and fat contents.

#### 2.2.3. Curcumin Stabilization Strategies for Chicken Preservation

Chicken meat is highly susceptible to microbial contamination and quality deterioration during refrigerated storage because of its high moisture content, nutrient-rich composition, and short shelf life. Compared with pork and beef, available chicken studies place less emphasis on freshness indication and more on stabilizing curcumin before or during incorporation into packaging matrices. Cyclodextrin inclusion and metal-coordinated hydrogel networks have been used to increase curcumin compatibility, stability, barrier performance, antioxidant activity, antibacterial activity, and blue-light-responsive function [44,45]. When applied to chicken meat, these systems reduce microbial growth, lipid oxidation, TVB-N formation, and pH increase while helping maintain color and texture. Current chicken-related studies therefore point to a design logic centered on curcumin stabilization and matrix integration. Future work should move beyond proof-of-concept formulations and test these materials under realistic poultry packaging conditions, including variable surface moisture, mixed spoilage microbiota, retail lighting, and scale-up feasibility.

### 2.3. Seafood

Seafood is among the most perishable food categories. This is mainly due to its high-water activity, abundant nutrients, fragile tissue structure, and, in many species, high levels of polyunsaturated lipids. These characteristics accelerate microbial proliferation, lipid oxidation, protein degradation, discoloration, texture softening, and off-odor formation during chilled storage. Compared with terrestrial meats, seafood spoilage is more closely associated with psychrotrophic bacteria, endogenous enzymatic activity, and rapid accumulation of nitrogenous metabolites such as TVB-N, trimethylamine, and biogenic amines. These characteristics make seafood an important field for curcumin-based preservation. Curcumin can contribute antioxidant protection, microbial inhibition, light-activated photodynamic inactivation, and pH-responsive color changes related to volatile amine accumulation. Direct use of free curcumin remains inefficient because of poor aqueous solubility, limited chemical stability, and weak compatibility with wet seafood packaging environments. Recent studies have therefore incorporated curcumin into nanocarriers, protein-based delivery systems, metal–organic frameworks, polysaccharide–protein films, hydrogels, and photodynamic formulations to improve dispersion, regulate activation, and align curcumin function with seafood-specific spoilage pathways.

#### 2.3.1. Curcumin-Based Preservation of Fish Products

Within seafood, fish products have received the most attention in curcumin-based preservation studies because their quality loss reflects major aquatic spoilage pathways, including microbial growth, lipid oxidation, protein degradation, water migration, and volatile nitrogen accumulation. Curcumin-mediated photodynamic inactivation is attractive for fish because it can reduce microbial contamination without thermal damage. In salmon and large yellow croaker, curcumin-based PDI inhibited pathogens or spoilage bacteria and slowed pH increase, TVB-N formation, lipid deterioration, protein degradation, color change, and moisture loss [4,46]. Combination with ε-polylysine hydrochloride enhanced bacterial damage, while molecular modification of curcumin improved water solubility and photosensitizing efficiency [46,47]. These studies suggest that curcumin-based PDI is effective only when curcumin remains dispersed, photoactive, and sufficiently close to microbial cells on moist fish surfaces.

Carrier engineering has therefore become central to translating curcumin photosensitization into more stable fish preservation systems. Nano-silica, Antarctic krill protein-based carriers, curcumin–protein conjugates, MOF-containing hydrogels, and polysaccharide–protein films have been used to enhance curcumin loading, water dispersibility, photostability, release behavior, barrier performance, and spoilage-related color response [10,48,49,50,51]. These examples mark a shift from free-curcumin treatment toward carrier-assisted designs that coordinate solubility enhancement, photostability, controlled release, barrier reinforcement, and freshness sensing. They also show that the carrier is not a passive support but a determinant of whether curcumin can remain active in wet fish systems.

For fish fillets, curcumin-based films increasingly combine active preservation with structural reinforcement and, in some cases, intelligent monitoring. Nanoparticle-loaded films, PVA/chitosan films, bilayer films, responsive nanofibers, and plasma-modified chitosan films have been applied to different fish fillets to delay microbial growth, TVB-N accumulation, lipid oxidation, texture loss, and sensory deterioration [52,53,54,55,56]. Although their material designs differ, they share the same objective: improving curcumin stability, release, barrier performance, and surface activity under high-moisture seafood conditions. Fish-related studies therefore show a clear progression from curcumin as a single active additive toward engineered preservation interfaces based on nanoparticles, protein conjugates, MOFs (metal–organic frameworks), bilayer films, hydrogels, and modified polymer matrices. The remaining challenge is to validate whether these materials retain sufficient curcumin activity, light responsiveness, and color reliability under realistic fish packaging conditions.

#### 2.3.2. Protein-Assisted Curcumin Systems for Shrimp Preservation

Shrimp is more moisture-sensitive and enzyme-active than many finfish, so its quality loss is governed not only by external microbial contamination but also by endogenous enzymatic reactions, protein structural disruption, lipid oxidation, and water migration. Current curcumin-based work on shrimp follows a design logic similar to salmon studies, but with stronger emphasis on stabilizing curcumin through marine protein carriers before photodynamic use. Soluble Antarctic krill protein–curcumin complexes improved curcumin stability and enabled more effective PDI against native shrimp microflora, while reducing TVB-N and malondialdehyde formation, suppressing endogenous enzyme activity, and preserving myofibrillar integrity [57]. These effects are important because shrimp texture deterioration is closely associated with muscle fiber damage and water loss. Evidence remains limited, however, and further studies should examine whether protein-based PDI formulations remain robust across different shrimp products, microbial loads, freezing histories, and industrial light-exposure conditions.

### 2.4. Other Food Matrices

Beyond fruits, meats, and seafood, curcumin-based preservation has been explored in edible fungi, mold-prone agricultural commodities, and liquid foods. These applications are less numerous but illustrate the adaptability of curcumin when its delivery form matches the dominant spoilage pathway. For fresh Tremella fuciformis, curcumin-mediated photodynamic inactivation provided a non-thermal strategy for reducing bacterial growth while helping retain appearance-related quality during storage [12]. In peanuts, cinnamaldehyde–curcumin Pickering emulsions improved the stability and delivery of hydrophobic antimicrobials, combining curcumin-mediated photodynamic activity with cinnamaldehyde-related antimicrobial effects [1]. For milk preservation, curcumin-loaded metal–phenolic nanoparticles incorporated into chitosan films improved film compatibility, mechanical strength, barrier performance, UV shielding, antioxidant activity, and antibacterial effects, thereby delaying milk spoilage [11]. These studies suggest that curcumin can be adapted beyond solid fresh foods through photodynamic decontamination, emulsion-based hydrophobic antimicrobial delivery, and nanocomposite film design. However, these applications remain largely proof-of-concept, and further work is needed to assess sensory impact, migration behavior, light-exposure feasibility, and performance under realistic storage and distribution conditions.

## 3. Anthocyanin-Based Active and Intelligent Applications

**Figure 3:**
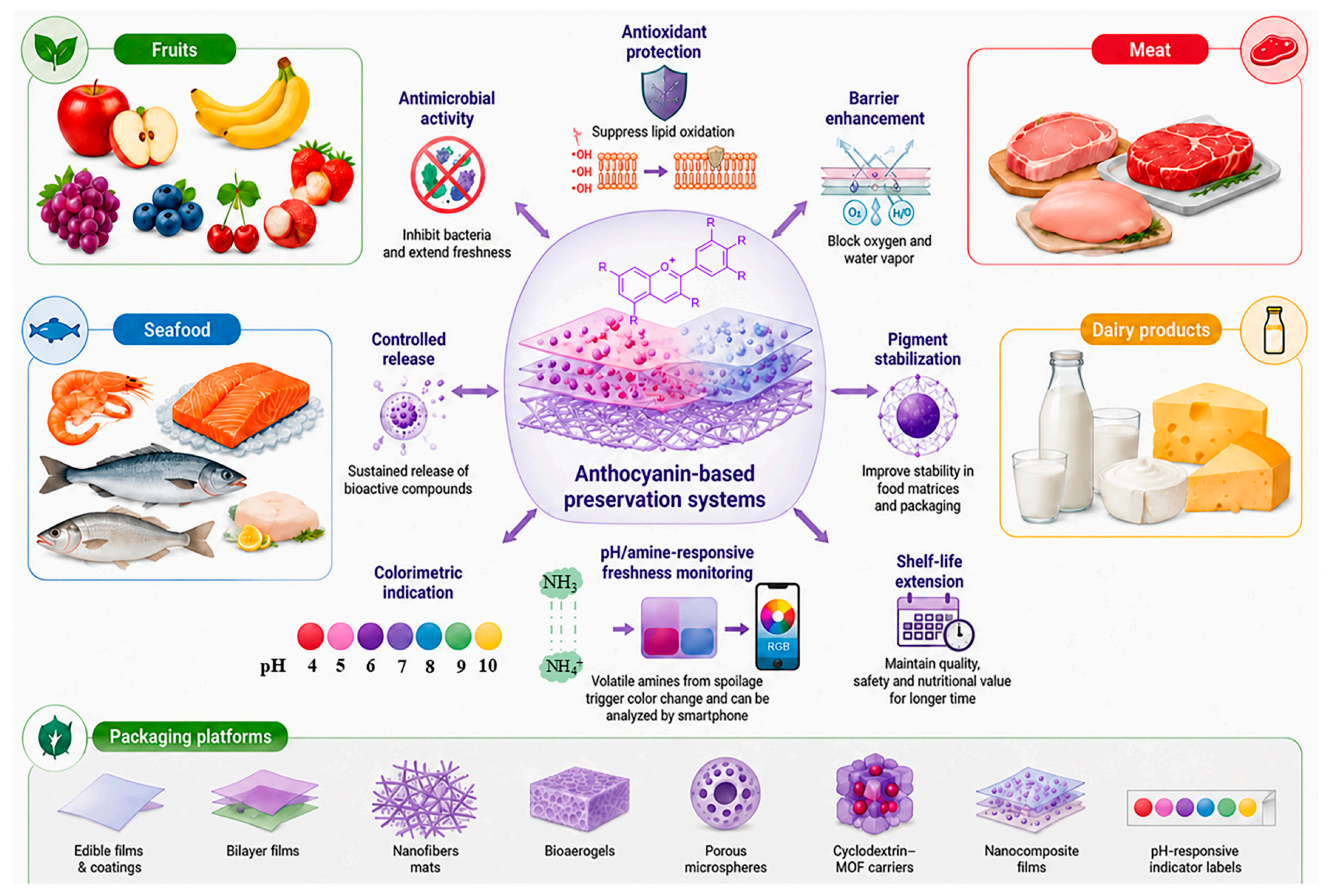
Overview of anthocyanin-based active and intelligent food applications: pH- and amine-responsive colorimetric monitoring, pigment stabilization, antimicrobial support, and matrix-dependent indicator reliability.

Building on the curcumin-based systems discussed above, anthocyanins represent another major class of natural pigments for active and intelligent food applications. Anthocyanins are water-soluble pigments widely distributed in fruits, vegetables, flowers, and plant-derived by-products, and their value in food systems derives from antioxidant activity, antimicrobial potential, and pronounced pH-dependent color transitions. Compared with curcumin formulations, which often require hydrophobic carrier engineering, photostability enhancement, or photodynamic activation, anthocyanin-based materials are more closely associated with visible freshness indication. Their molecular structures can reversibly shift among differently colored forms under changing pH conditions. Therefore, spoilage-related pH shifts, volatile amine accumulation, protein degradation, or acidity changes can be translated into readable color signals. However, anthocyanin color changes should not be interpreted as universal freshness signals, because they primarily reflect the local acid–base environment around the indicator rather than the full microbiological, oxidative, enzymatic, or sensory status of the food. The same color transition may therefore have different meanings in different matrices: alkaline amine accumulation is more relevant to meat and seafood spoilage, whereas acidification is more important in many dairy systems, and fruit systems may be affected by intrinsic acidity, respiration, surface moisture, and background color. Thus, anthocyanin-based indicators require food-specific calibration against appropriate quality indices, such as pH, TVB-N, microbial counts, volatile acids, lipid oxidation markers, and sensory acceptability. Free anthocyanins, however, are difficult to apply directly because they are sensitive to light, heat, oxygen, enzymes, metal ions, and alkaline environments. Their color output may also be affected by food composition, packaging atmosphere, polymer interactions, and coexisting active compounds. Recent studies have therefore incorporated anthocyanins into films, coatings, nanofibers, hydrogels, aerogels, multilayer structures, metal–organic frameworks, cyclodextrin-based carriers, and related composite formats (Figure 3). These designs seek to preserve pigment integrity while improving barrier performance, mechanical strength, release behavior, and indicator reliability. Therefore, this section distinguishes the sensing role of anthocyanins from the preservation functions provided by the surrounding material system.

### 3.1. Fruits

#### 3.1.1. Anthocyanin-Based Packaging for Climacteric Fruits

In climacteric fruit systems, anthocyanin-containing materials have mainly been developed as integrated packaging components rather than independent preservation agents. For fresh-cut apples, elderberry anthocyanins in konjac glucomannan/polyvinylpyrrolidone fiber films contributed antioxidant and antibacterial functions, while matrix interactions improved film stability, water resistance, and mechanical integrity [58]. The resulting films mitigated enzymatic browning, moisture loss, texture decline, and microbial contamination, which are major deterioration pathways in fresh-cut apples. For whole bananas, radish anthocyanins were incorporated into an agar-based bilayer film with neem essential oil and TiO_2_ [59]. Anthocyanins supplied antioxidant and color-related functions, whereas the essential oil and inorganic filler mainly strengthened antimicrobial and barrier properties. These examples show that anthocyanin-based climacteric-fruit systems are highly formulation-dependent: their effectiveness arises from coordination between pigment stability, polymer compatibility, barrier regulation, and auxiliary active components. The role of anthocyanins should therefore be separated from that of essential oils, inorganic fillers, and the structural matrix.

#### 3.1.2. Anthocyanin-Based Systems for Non-Climacteric Fruits

For non-climacteric fruits, anthocyanin-related systems are generally directed toward controlling surface microbial decay, water loss, softening, oxidative deterioration, and visual quality changes, rather than modulating respiration-driven ripening. Strawberry has been a common model because its fragile tissue, high water activity, and susceptibility to microbial spoilage lead to rapid postharvest quality loss. Anthocyanin-containing films combined with silver nanoparticles, related polyphenols, essential oils, or peptides have been used to integrate pH-sensitive color response, antioxidant activity, antibacterial protection, and quality retention [2,60,61]. Although proanthocyanidin is not an anthocyanin pigment in the strict sense, its inclusion is informative because it shows how related polyphenols can reinforce bio-based packaging when paired with hydrophobic antimicrobials and crosslinked polysaccharide networks [60]. These strawberry studies underline the importance of co-formulation: pH-responsive pigments require stabilizing matrices and complementary antimicrobial or reinforcing agents to maintain both visual responsiveness and packaging performance.

For grapes and blueberries, the design focus shifts toward barrier regulation, pigment stabilization, and intelligent response in packaging formats suitable for berry-like fruits. Flexible polysaccharide films, cyclodextrin–metal–organic framework encapsulation, and electrospun nanofibers have been used to improve anthocyanin stability, flexibility, water resistance, oxygen barrier capacity, antimicrobial activity, and pH-responsive color changes [62,63,64]. These systems also reveal important trade-offs. Polysaccharide-rich films may suffer from humidity sensitivity, while porous nanofibers may improve loading and response but weaken stiffness or gas-barrier performance. Thus, berry-fruit systems need to balance pigment accessibility, structural stability, gas/moisture regulation, and readable color transitions.

Beyond exogenous packaging, postharvest litchi provides a distinct example in which anthocyanins are involved through endogenous metabolic regulation rather than direct incorporation into packaging materials. Litchi pericarp browning is closely associated with anthocyanin degradation and altered phenolic metabolism. Exogenous inoculation with Pediococcus pentosaceus SSC12 alleviated browning by regulating anthocyanin biosynthesis, transport, and degradation-related pathways [65]. This study differs from film-based approaches in which anthocyanins are used as indicators or additives, because native anthocyanins are treated as intrinsic quality markers whose metabolic stability can be biologically modulated after harvest. It therefore broadens anthocyanin-based applications from packaging formulation to physiological quality regulation. Evidence from non-climacteric fruits suggests that recent strategies increasingly combine visual freshness indication, moderate active protection, and pigment stabilization. Their reliability remains matrix-dependent, since fruit acidity, surface moisture, microbial ecology, polymer–pigment interactions, and storage humidity can all influence protective effects and colorimetric accuracy.

### 3.2. Meats

#### 3.2.1. Anthocyanin-Based Indicators for Pork Freshness Monitoring

In pork packaging, anthocyanin-based films are mainly designed for pH/amine-responsive freshness indication, with active functions added to limit microbial growth, oxidation, and spoilage volatiles. Pork deterioration is associated with pH increase, TVB-N accumulation, color changes, and microbial succession, so anthocyanins are most valuable when these changes can be converted into visible or digitally readable signals. Compared with their preservative role, the most consistent function of anthocyanins in pork systems is freshness indication; their direct contribution to microbial inhibition or oxidation control is less certain because many formulations contain additional active agents. Nanomaterial reinforcement and encapsulation have been used to improve mechanical strength, barrier properties, antioxidant activity, heat/light stability, and colorimetric durability [66,67]. However, filler selection must consider both structural performance and indicator compatibility, because some nanomaterials may interfere with anthocyanin color response. These findings highlight the need to protect anthocyanins while maintaining their exposure to spoilage-related volatiles. This creates a central design trade-off, because strong encapsulation or dense reinforcement may improve pigment stability but reduce the accessibility of volatile amines, whereas highly exposed pigments may respond rapidly but degrade more easily during storage.

Anthocyanins have also been combined with natural antimicrobials, polyphenols, or porous carriers to align freshness monitoring with quality protection. Tea polyphenols, essential oils, and ZIF-based antimicrobial carriers have been used to stabilize anthocyanins, suppress microbial growth, delay off-odor formation, and maintain visible pH/NH_3_ responses during pork storage [68,69,70]. These systems show that anthocyanins function more convincingly as intelligent indicators when supported by agents that reduce microbial pressure and stabilize the packaging microenvironment. Therefore, shelf-life extension in these systems should be attributed to the integrated formulation unless appropriate controls separate anthocyanin effects from those of tea polyphenols, essential oils, ZIF-based carriers, or polymer matrices.

Co-pigmentation, multilayer construction, and host–guest encapsulation have been further used to improve anthocyanin stability and monitoring reliability. Polylysine, catechin, ferulic acid, carbon dots, and cyclodextrin–metal–organic frameworks have been used to enhance pigment retention, light stability, antimicrobial performance, reversible pH response, and smartphone-based freshness analysis [71,72,73,74,75]. Although procyanidin-Zn^2+^ metal–polyphenol networks are not anthocyanin indicators, they provide a related example of how polyphenol coordination can strengthen active pork packaging [74]. Across these studies, a common conclusion is that anthocyanin indicators require stabilization, whereas the unresolved issue is whether these stabilization strategies alter signal accuracy. Co-pigmentation and host–guest encapsulation can improve color stability, yet they may also alter the initial color, response range, or reversibility of the indicator. Taken together, pork studies suggest that future designs should prioritize pigment stability, amine accessibility, reduced matrix interference, and quantitative links between color changes and real quality indices. More importantly, color changes should be calibrated against TVB-N, microbial counts, pH, and sensory acceptability in the same pork system, because visual response alone cannot confirm whether the indicator accurately distinguishes freshness, sub-freshness, and spoilage.

#### 3.2.2. Anthocyanin-Based Colorimetric Monitoring of Beef Freshness

In beef packaging, anthocyanin-based systems place greater emphasis on volatile amine recognition, sub-freshness discrimination, and quantitative color analysis, while active protection is usually introduced to support the reliability of the indicator response. This creates a useful transition from pork systems, where formulation design often aims to balance antimicrobial action and signal retention, to beef systems, where the accuracy and grading capacity of the color response become more prominent. Co-pigmented double-layer films, pH-adjusted chitosan/gelatin films, and tea polyphenol-containing polysaccharide films have been used to improve color intensity, amine-response sensitivity, antioxidant activity, antibacterial performance, and smartphone-assisted RGB evaluation [5,76,77,78]. These systems show that anthocyanins are most useful when pH/amine-responsive behavior is supported by co-pigmentation, matrix calibration, and active barrier design. Moisture sensitivity, initial color selection, pigment stability, and the robustness of RGB-based models remain key issues under variable retail conditions.

### 3.3. Seafood

#### 3.3.1. Anthocyanin-Based Monitoring of Fish Freshness

Moving from terrestrial meats to seafood, the role of anthocyanins becomes even more closely tied to volatile nitrogen accumulation because fish spoilage is strongly associated with TVB-N, ammonia, trimethylamine, lipid oxidation, moisture migration, and off-odor formation. In fish systems, anthocyanin-based packaging is therefore mainly oriented toward pH/NH_3_-responsive freshness indication, while antibacterial and antioxidant components are introduced to support the reliability of the signal. Salmon has been a representative model for testing whether colorimetric response can be integrated with active quality protection. Anthocyanin-containing labels and films combined pH/ammonia-sensitive color changes with antibacterial and antioxidant functions by using porous carriers, essential oils, or phenolic stabilizers [79,80]. These studies show that the reliability of salmon freshness labels depends less on anthocyanin incorporation alone than on stabilizing the pigment and tuning the film microenvironment so that mid-stage spoilage can be recognized before obvious sensory rejection.

For other fish products, multilayer structures and sustainable polysaccharide matrices further illustrate the shift from simple indicators to active-intelligent systems. Double-layer films and polysaccharide–anthocyanin films have been used to combine ammonia-responsive color change, antimicrobial support, oxygen barrier properties, and oxidation control [81,82]. Related polyphenol-crosslinked films, although not anthocyanin-based indicators, further demonstrate the relevance of controlled antioxidant release in fish packaging because lipid oxidation remains a major deterioration route [83]. Collectively, fish studies suggest that anthocyanin systems are most persuasive when pigment stabilization, amine accessibility, antimicrobial support, and oxidation control are considered together. Practical application still requires closer attention to humidity, light exposure, species-dependent spoilage profiles, and quantitative relationships between color changes and established freshness indices.

#### 3.3.2. Anthocyanin-Based Indicators for Shrimp Spoilage Detection

Shrimp is a particularly suitable matrix for anthocyanin-based freshness monitoring because its spoilage is rapidly accompanied by microbial growth, pH elevation, volatile amine accumulation, and TVB-N formation. Compared with fish, shrimp applications place stronger emphasis on fast response to alkaline spoilage volatiles and on maintaining pigment activity in highly moist matrices. Recent studies therefore focus less on anthocyanins as direct preservatives and more on their role as pH/amine-responsive indicators supported by antibacterial or antioxidant components. Bioaerogels, nanocomplexes, cellulose nanocrystal-reinforced films, and inorganic filler-containing films have been used to stabilize anthocyanins, improve barrier and mechanical properties, retain antioxidant activity, and maintain visible responses to shrimp spoilage [84,85,86,87]. These examples indicate that shrimp packaging requires a careful balance between pigment protection and analyte accessibility, since excessive encapsulation or dense matrices may stabilize anthocyanins but slow their response to volatile amines.

Other studies have strengthened shrimp packaging by combining anthocyanins with antimicrobial peptides, polyphenols, or photocatalytic bilayer structures. Mixed anthocyanins, nisin, tea polyphenols, and photocatalytic layers have been used to integrate readable spoilage signals with antibacterial activity, antioxidant protection, pigment stabilization, or visible-light-assisted function [88,89,90]. However, the limited extension of shrimp shelf life in some systems suggests that stabilizing the colorant does not automatically translate into strong preservation efficacy. This body of work reveals a clear movement toward integrated shrimp-packaging systems in which anthocyanins provide readable spoilage signals, while nisin, tea polyphenols, nanofillers, or photocatalytic layers support microbial control and pigment stability. Future work should place greater emphasis on response kinetics, humidity tolerance, migration safety, and quantitative calibration among color changes, TVB-N, microbial counts, and sensory acceptability.

### 3.4. Dairy Products

Anthocyanin-based applications in this section are mainly represented by dairy products. Unlike meat and seafood, dairy spoilage often involves acidification, microbial proliferation, and increased acidity; therefore, anthocyanin indicators must be calibrated to pH decline and volatile acid formation rather than alkaline amine accumulation. In milk and cheese systems, anthocyanins have been incorporated into emulsion films, polysaccharide films, nanoparticle-reinforced films, and bilayer structures to combine pH/volatile-acid sensitivity with antimicrobial activity, antioxidant capacity, UV shielding, color stability, controlled release, and moisture resistance [91,92,93,94]. Dairy-related studies indicate that anthocyanins can provide useful pH-responsive freshness information when supported by co-pigments, essential oils, nanoparticles, or bilayer structures. Their reliability, however, remains product-specific because milk opacity, protein and fat interactions, acidity range, moisture transfer, storage temperature, migration safety, pigment stability, and sensory effects from essential oils can all interfere with color development and signal interpretation.

## 4. Emerging Carotenoid-Based Active Applications

**Figure 4:**
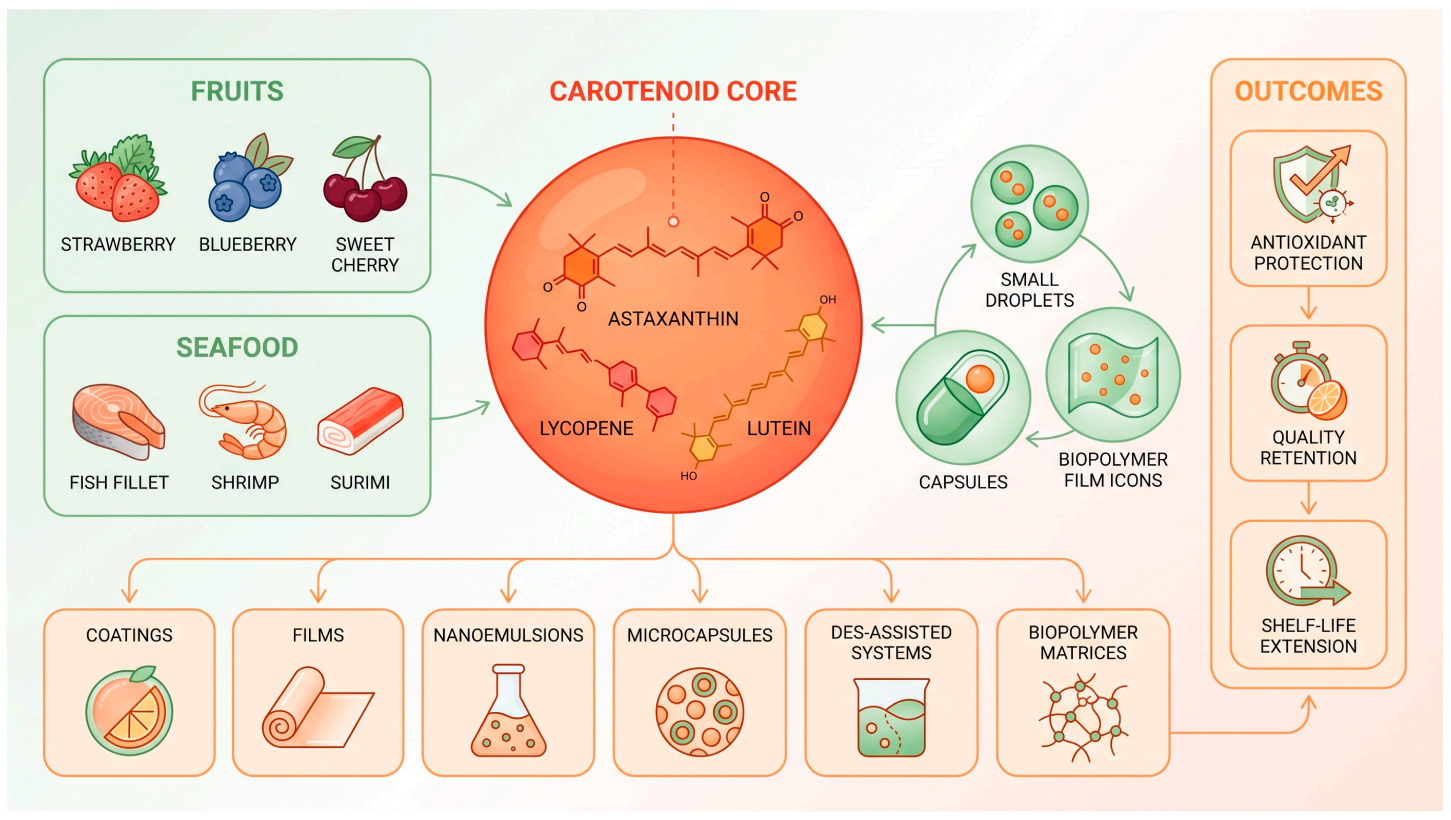
Carotenoid-based active preservation systems and stabilization strategies for oxidation-sensitive foods: antioxidant protection, carrier-assisted dispersion, microencapsulation, emulsion delivery, and by-product valorization.

Carotenoids, such as astaxanthin, lutein, and lycopene, represent a third group of natural pigments with potential value in active food preservation. They are lipid-soluble compounds characterized by extended conjugated double-bond systems, which give them strong antioxidant capacity and enable them to quench reactive oxygen species and delay oxidative deterioration. Compared with the curcumin- and anthocyanin-based systems discussed above, carotenoid-based applications remain less extensively investigated in food preservation. This is partly because carotenoids are highly hydrophobic, sensitive to light, oxygen, and heat, and generally less suitable for direct pH-responsive freshness indication. As a result, current studies have focused mainly on their use as antioxidant-dominant active agents rather than as intelligent colorimetric indicators. Existing strategies usually involve incorporating carotenoids into lipid-compatible films, emulsions, nanoparticles, biopolymer matrices, or other protective carriers to improve dispersion, limit pigment degradation, and enhance delivery in food systems (Figure 4). Although the available evidence is still limited, carotenoids have considerable research potential, especially for lipid-rich or oxidation-prone foods where oxidative stability is a major determinant of quality loss. Further development of carrier-assisted stabilization, controlled release, and matrix-compatible packaging designs may expand their role from simple antioxidant additives to more effective multifunctional preservation systems.

### 4.1. Fruits

Current carotenoid-based fruit preservation studies are relatively limited and focus mainly on highly perishable small fruits, especially strawberries, blueberries, and sweet cherries. In these systems, carotenoids are used less as freshness indicators and more as antioxidant-active compounds that require carrier assistance to overcome poor water compatibility and environmental instability. For strawberry preservation, astaxanthin has been incorporated into sodium alginate coatings using deep eutectic solvent-assisted formulation and into pullulan/gellan gum films through nanoemulsion loading [3,95]. These approaches improved astaxanthin incorporation into hydrophilic biopolymer systems and delayed quality deterioration, weight loss, and microbial or visual quality decline. Their broader significance lies in showing that astaxanthin performance depends less on direct pigment addition than on carrier-assisted stabilization and delivery. Astaxanthin-based strawberry preservation therefore illustrates a recurring principle for carotenoid systems: practical efficacy requires adequate dispersion, protection against degradation, and compatibility with the biopolymer matrix.

Lycopene-based fruit packaging follows a similar design logic, with microencapsulation acting as the key strategy for converting an unstable lipophilic pigment into a usable packaging ingredient. In blueberry and sweet cherry preservation, lycopene microcapsules improved pigment dispersion within hydrophilic matrices and contributed to antioxidant, UV-blocking, antibacterial-supporting, and quality-retention functions [14,15]. These studies suggest that lycopene has potential in fruit preservation, but mainly when it is converted from a light- and oxygen-sensitive hydrophobic pigment into a protected microencapsulated component. Taken together, carotenoid-based fruit applications remain at an early stage compared with curcumin and anthocyanin systems. Their strongest contribution lies in antioxidant protection and light/oxygen-related quality maintenance, whereas their roles in antimicrobial control and intelligent freshness indication are still less developed. Future work should therefore focus on carrier durability, release behavior, sensory influence, migration safety, and validation across broader fruit types under realistic storage conditions.

### 4.2. Seafood

#### 4.2.1. Carotenoid-Related Quality Control in Fish Products

In fish systems, carotenoid-related applications are still sparse and differ from the packaging-oriented strategies commonly reported for curcumin and anthocyanins. The available studies suggest two distinct routes: incorporation into processed fish matrices to improve product stability, and dietary supplementation before harvest to enhance intrinsic muscle quality. Unlike curcumin and anthocyanins, carotenoids currently lack a well-established postharvest packaging role in fish; the evidence is fragmented and does not yet support a general conclusion that carotenoids function as standalone freshness-preserving agents. In printed surimi, lutein was introduced into a Ca^2+^-nano starch-lutein system, where the matrix mainly contributed to printability and gel reinforcement, while lutein-related functionality supported fresh-keeping by helping suppress bacterial growth and amino acid decomposition [96]. This study links carotenoid incorporation with 3D-printed functional foods, but it also reveals a limitation: freshness deterioration was not visually distinguishable from the surimi matrix itself. The separate use of an anthocyanin-based indicator therefore shows a clear division of function: lutein contributed to active preservation within the printed surimi matrix, whereas anthocyanins provided the pH/ammonia-responsive color signal. Thus, carotenoids in this context should not be interpreted as intelligent indicators, but as active components embedded within a broader active-intelligent printing platform. This functional separation is important because it prevents over-attribution, as the intelligent monitoring function in this system comes from anthocyanins rather than from lutein.

A different perspective is provided by dietary xanthophyll supplementation in southern catfish [97]. Rather than acting after processing or packaging, xanthophylls were used to regulate fish quality during aquaculture. Appropriate supplementation enhanced growth, antioxidant status, immune response, pigmentation, and several meat-quality traits related to water retention and texture. Although this study is not a postharvest preservation intervention, it is still relevant because improved antioxidant capacity, muscle structure, and water-holding properties may influence the subsequent quality stability of fish products. Its implication for preservation is therefore indirect: carotenoids may strengthen the initial quality and oxidative resilience of fish muscle before processing, but their protective effects during storage still require separate validation. Together, these fish-related studies indicate that lutein and xanthophylls have potential in seafood quality control, but current applications remain fragmented. Their value lies more in structural reinforcement, antioxidant support, and preharvest quality modulation than in direct freshness indication or standalone preservation. The current controversy is therefore not whether carotenoids have antioxidant potential, but whether this potential can be translated into measurable postharvest preservation under realistic fish storage conditions. At present, fish studies provide indirect or matrix-specific evidence rather than a coherent carotenoid-based preservation strategy.

#### 4.2.2. Astaxanthin-Based Active Films for Shrimp Preservation

Compared with fish, shrimp provides a more direct example of carotenoid-based active packaging. Astaxanthin extracted from shrimp-shell by-products using deep eutectic solvents was incorporated into chitosan films together with DES- and PEG-related plasticizing strategies [6]. Here, DES should be understood as deep eutectic solvents rather than a separate preservative mechanism. This work is notable because it connects valorization of seafood processing waste with the development of biodegradable active packaging for perishable seafood, two objectives that are often treated separately. In the film matrix, DES improved plasticization and facilitated the incorporation of hydrophobic astaxanthin, chitosan provided the film-forming backbone and intrinsic antimicrobial contribution, and astaxanthin supplied antioxidant biofunctionality. Therefore, the preservation effect observed in shrimp should be understood as the outcome of a chitosan–DES–astaxanthin system rather than astaxanthin alone. This makes the shrimp study more directly relevant to active packaging than the fish examples, but it also introduces the same attribution challenge observed in other pigment-based systems: chitosan, DES-assisted plasticization, and astaxanthin may all contribute to the final preservation outcome.

When applied to shrimp, the DES-Ast-Ch coating reduced microbial development, weight loss, and TVB-N accumulation, indicating better freshness retention during storage [6]. More importantly, this study illustrates a practical direction for carotenoid-based seafood packaging: lipid-soluble pigments recovered from seafood by-products can be reintegrated into biodegradable matrices designed for similarly perishable seafood products. This circular-use logic gives astaxanthin-based packaging additional value beyond its antioxidant function. Nevertheless, the system remains at an early stage. Further work is needed to clarify astaxanthin migration, sensory influence, long-term pigment stability, safety of DES-containing films, and performance under industrial storage and distribution conditions. Current evidence therefore supports astaxanthin as a promising antioxidant and antimicrobial-supporting component for shrimp packaging, but commercial relevance will depend on whether the integrated film system remains stable, safe, and effective beyond laboratory-scale preservation tests. Overall, seafood evidence suggests that carotenoids are better suited to antioxidant-oriented preservation than to freshness indication, although their specific contribution still requires validation using appropriate carrier and matrix controls.

## 5. Comparative Assessment of Curcumin, Anthocyanins, and Carotenoids

Curcumin, anthocyanins, and carotenoids are often grouped together as natural pigments, but their roles in food systems are not equivalent. The differences come first from their chemical structures. Curcumin contains phenolic and conjugated diketone structures that support antioxidant, antimicrobial, and light-activated photodynamic activity. Anthocyanins undergo pH-dependent structural transformation, which makes them particularly useful for colorimetric responses to acidity changes or volatile amines. Carotenoids, with long conjugated polyene chains, are better suited to quenching reactive oxygen species and slowing lipid oxidation. These structural differences explain why curcumin is more often used for active preservation, anthocyanins for freshness monitoring, and carotenoids for antioxidant-oriented protection. At the same time, the actual preservation outcome is rarely produced by the pigment alone. Carrier materials, film matrices, controlled-release systems, and coactive compounds often determine whether the pigment remains stable, accessible, safe, and effective in real food environments.

### 5.1. Structure-Dependent Functional Mechanisms of Natural Pigments

**Figure 5:**
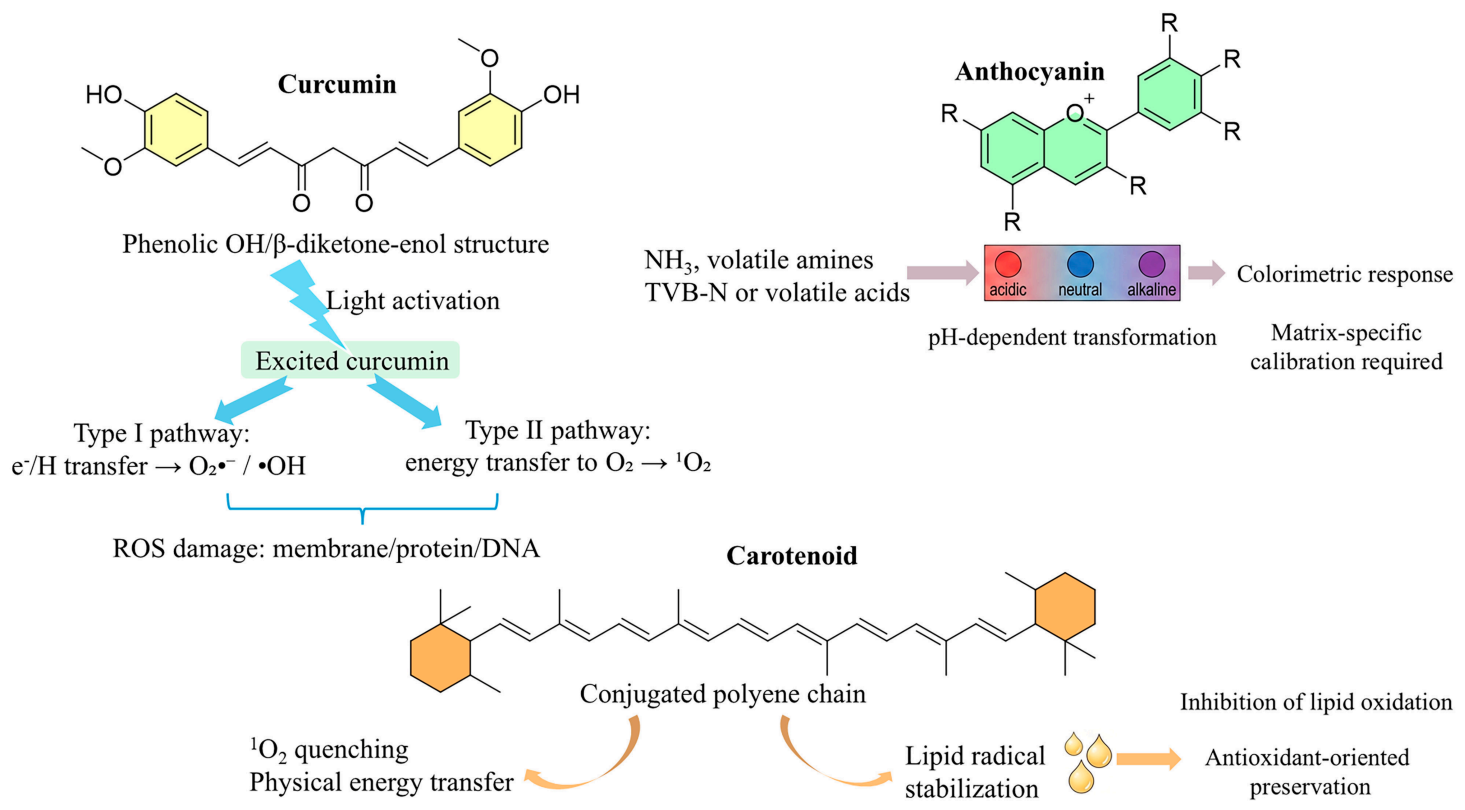
Structure-dependent mechanisms of curcumin, anthocyanins, and carotenoids in antioxidant preservation, photodynamic inactivation, and freshness monitoring.

Curcumin, anthocyanins, and carotenoids show different functions largely because their molecular structures favor different reaction pathways (Figure 5). Curcumin contains phenolic aromatic rings and a conjugated β-diketone/enol structure, allowing hydrogen or electron donation for radical scavenging; the resulting radical intermediates can be stabilized by the conjugated system, helping interrupt oxidative chain reactions. The same conjugated structure also enables visible-light absorption and photodynamic activity. After photoactivation, curcumin can enter an excited state and transfer electrons or hydrogen atoms to surrounding substrates through a Type I pathway, or transfer energy to molecular oxygen through a Type II pathway, generating reactive oxygen species such as superoxide radicals, hydroxyl radicals, and singlet oxygen. These ROS damage microbial membranes, proteins, and nucleic acids, which explains its use in photodynamic inactivation of Listeria monocytogenes on salmon and fresh-cut pears [4,19]. Its hydrophobic backbone may also interact with lipid-rich microbial membranes, increasing permeability, disturbing membrane integrity, and promoting leakage of intracellular components. However, poor water solubility and environmental instability mean that these functions usually require carrier-assisted dispersion and protection.

Anthocyanins are based on a flavylium structure that undergoes pH-dependent transformation among different colored forms. This explains their frequent use in freshness monitoring, especially in meat and seafood where volatile amines, ammonia, and TVB-N increase local alkalinity and trigger visible color changes [5,79,84]. Their phenolic hydroxyl groups may also contribute to antioxidant activity through radical scavenging, but direct antimicrobial preservation is often difficult to separate from the effects of co-formulated essential oils, tea polyphenols, peptides, nanofillers, or polymer matrices. Moreover, the color response reflects local pH or amine chemistry rather than freshness in a universal sense, so anthocyanin indicators must be calibrated for each food matrix, including acidifying systems such as dairy products [91,92,93,94]. Carotenoids, by contrast, are dominated by long conjugated polyene chains that quench singlet oxygen by physical energy transfer and stabilize lipid radicals through electron delocalization, making them more suitable for antioxidant-oriented preservation than colorimetric indication. Astaxanthin, lycopene, and lutein have therefore been applied mainly to oxidation-sensitive fruits, shrimp, and surimi systems [3,6,14,15,96]. These differences also explain why the three pigments are not interchangeable in packaging design. Curcumin is particularly relevant when antioxidant activity, microbial control, or light activation is needed; anthocyanins are most informative when spoilage produces measurable pH or amine changes; and carotenoids are more suitable when oxidative deterioration, especially lipid oxidation, is the main quality-limiting factor.

### 5.2. Comparative Preservation and Monitoring Performance in Food Systems

As summarized in Table 1, no single pigment performs best across all food systems; performance depends on the dominant deterioration pathway, food matrix, and material design. It should be noted that the numerical values listed in Table 1 were obtained from different studies with different food matrices, pigment concentrations, carrier systems, storage temperatures, microbial loads, packaging formats, irradiation conditions, and evaluation endpoints. Therefore, these values are intended to provide representative performance ranges rather than direct head-to-head comparisons among pigment classes or carrier systems. Curcumin-based systems show the most consistent quantitative antimicrobial performance, particularly in photodynamic, controlled-release, and reinforced packaging formats. Representative studies reported banana and pork shelf-life extension of about 10 days, beef spoilage-bacteria inhibition of >95%, salmon bacteriostatic rates of approximately 99.99%, and salmon bacterial-count reduction of about 3.0 log CFU/g [4,16,31,41,49]. In fish systems, curcumin-based films extended grass carp shelf life to 10–14 days and Schizothorax fish fillet shelf life to 15 days [51,52]. These results suggest that curcumin is especially effective for antimicrobial preservation when its poor solubility and instability are addressed by photodynamic platforms, nanofibers, cyclodextrins, MOFs, protein carriers, or reinforced biopolymer matrices.

Anthocyanins show a different performance profile, functioning more reliably as freshness-monitoring pigments than as primary antimicrobial agents. Their pH- or amine-responsive color changes enabled visual or intelligent monitoring in strawberries, pork, shrimp, and fish, while reported preservation effects ranged from short-term extension of several hours to several days, and in some cases shelf-life doubling or extension from 6 to 12 days [2,68,75,80]. However, these effects often involved essential oils, tea polyphenols, antimicrobial peptides, nanofillers, or multilayer structures, making the anthocyanin-specific contribution difficult to isolate. Carotenoids remain less studied but are useful for oxidation-sensitive foods: astaxanthin extended strawberry shelf life from 12 to 18 days, lycopene-based systems preserved cherries for 15 days, and lutein contributed to freshness monitoring in surimi [15,95,96]. Overall, based on the currently reported study-specific evidence, curcumin-based systems appear more frequently associated with antimicrobial preservation, anthocyanin-based systems with freshness indication, and carotenoid-based systems with antioxidant-oriented quality protection. This trend should be interpreted as a functional tendency rather than a universal performance ranking.

### 5.3. Pigment Activity, Carrier Contribution, and Synergistic Effects

Carrier materials are widely used in pigment-based food systems because free pigments often suffer from poor solubility, low stability, weak matrix compatibility, uncontrolled migration, and limited contact with food surfaces. In real preservation systems, the contributions of pigments and carriers vary. In some cases, the main function is pigment-driven, while the carrier mainly improves dispersion, stability, loading, release, or microbial contact. For example, curcumin-mediated photodynamic antibacterial activity depends on the photosensitizing property of curcumin, but protein emulsions, gelatin coatings, and nanoencapsulation systems are required to enhance its dispersity, photostability, bioavailability, and interaction with microorganisms [41,49]. Similarly, carotenoid systems mainly rely on the antioxidant activity of astaxanthin or lycopene, whereas nanoemulsions and microcapsules improve pigment retention, oxidative stability, and compatibility with hydrophilic matrices [6,95]. In other cases, preservation results from pigment–carrier–coactive-agent synergy. This is especially common in anthocyanin-based intelligent packaging, where anthocyanins mainly provide pH- or amine-responsive color signals, while shelf-life extension is often supported by tea polyphenols, essential oils, nisin, eugenol, AgNPs, ZIF-based carriers, or multilayer structures [68,75]. Therefore, the reported antimicrobial, antioxidant, or monitoring effects should be interpreted as combined material-system outcomes rather than as mono-pigment effects.

The carriers used for natural pigments can be broadly classified into natural biopolymer-based carriers and synthetic or engineered carriers. Natural polymers, such as chitosan, gelatin, starch, cellulose, pectin, sodium alginate, konjac glucomannan, pullulan, gellan gum, and protein-based matrices, are attractive because of their biodegradability, film-forming ability, biocompatibility, and potential edibility. They are suitable for clean-label and sustainable packaging, and some, such as chitosan or protein-based carriers, can also provide antimicrobial activity, adhesion, water retention, or interfacial stabilization. However, they often show high moisture sensitivity, weak mechanical strength, limited barrier performance, and batch-to-batch variability, which may affect pigment stability and sensing accuracy. In contrast, synthetic polymers and engineered carriers, including PVA, PLA, polyurethane, cyclodextrin-based systems, MOFs, nanofibers, nanoparticles, and responsive hydrogels, generally offer better structural tunability, mechanical strength, loading capacity, and controlled-release behavior, making them useful for pigment protection, sustained release, stimuli-responsive delivery, or enhanced photodynamic efficiency [8,31]. Nevertheless, these systems may raise concerns regarding migration safety, degradation products, carrier toxicity, environmental persistence, production cost, and regulatory acceptance. Therefore, the most effective carrier is not necessarily the most complex one, but the one that matches the pigment limitation, target function, and dominant deterioration pathway of the food matrix.

### 5.4. Regulatory and Commercialization Considerations

For commercialization, the use of natural pigments in food systems cannot be judged only by their natural origin or biological activity. A pigment that is acceptable as a food colorant or natural additive may still require separate safety evaluation when it is incorporated into active films, coatings, nanocarriers, MOFs, DES-assisted systems, or intelligent labels. The key issues include migration level, degradation products, carrier toxicity, exposure dose, sensory impact, and whether the material is intended to contact food directly. From a regulatory perspective, pigment-based active and intelligent packaging should be evaluated as food-contact materials rather than only as natural pigments or food additives. In the United States, food-contact substances are regulated by the FDA through existing provisions in Title 21 of the Code of Federal Regulations, the Food Contact Notification program, or threshold-of-regulation exemptions. In the European Union, such materials should comply with Regulation (EC) No 1935/2004 and GMP requirements under Regulation (EC) No 2023/2006; plastic components are further covered by Regulation (EU) No 10/2011, while active and intelligent materials are addressed by Regulation (EC) No 450/2009 and may require EFSA safety assessment. In China, relevant requirements include the GB 4806, GB 9685, and GB 31604 series. Migration testing should consider food simulants, contact conditions, overall/specific migration, degradation products, nanoparticles, solvents, and non-intentionally added substances. For example, curcumin-loaded metal–phenolic nanoparticles in chitosan films improved milk preservation, but such nanocomposite systems would still require migration and toxicological assessment before practical use [11]. Similarly, DES-assisted astaxanthin-chitosan films for shrimp preservation raise questions about residual solvent, pigment migration, and long-term stability [6]. For anthocyanin-based intelligent films, commercialization also depends on whether the color response is accurate, reproducible, and not misleading under real storage conditions; smartphone-assisted beef freshness monitoring illustrates the need for quantitative calibration rather than simple visual judgment [5]. Industrial translation also requires several practical considerations. These include raw-material availability, batch-to-batch variation, extraction and purification cost, compatibility with existing coating, casting, extrusion, or printing processes, and pigment stability during film processing and storage. Although natural pigments are attractive for clean-label packaging, their sensitivity to light, oxygen, pH, heat, and humidity may increase formulation complexity and production cost. Commercially feasible systems should therefore minimize expensive nanocarriers or complex multi-component designs unless they provide clear performance advantages under real processing and storage conditions. Overall, future pigment-based systems should be developed with food-contact safety, regulatory classification, scale-up stability, consumer acceptance, and validated real-food performance considered from the beginning.

## 6. Discussion and Future Perspectives

Current studies on curcumin, anthocyanins, and carotenoids demonstrate considerable potential, but several limitations remain. First, many systems are still proof-of-concept materials evaluated under simplified laboratory conditions, whereas validation in real food matrices, retail environments, and commercially relevant packaging scenarios remains insufficient. Second, component attribution remains difficult in complex formulations, because carriers and coactive additives may also affect antimicrobial activity, barrier performance, release behavior, and color stability. Third, pigment instability remains a shared challenge: curcumin is limited by poor solubility and photodegradation, anthocyanins by sensitivity to pH, light, oxygen, humidity, and matrix interference, and carotenoids by hydrophobicity, oxidation, and isomerization. In addition, long-term stability under practical storage and distribution conditions, including temperature fluctuation, light exposure, oxygen, high humidity, mechanical stress, and prolonged contact with food matrices, is still insufficiently evaluated. Reproducibility is another critical issue because pigment source, extraction method, purity, film thickness, polymer composition, drying conditions, and storage history can strongly affect release behavior, antimicrobial performance, and color response.

For commercial translation, future studies should move beyond short-term functional demonstrations and address manufacturing feasibility more critically. Industrial processing may involve casting, coating, extrusion, printing, lamination, drying, sealing, or sterilization, all of which can affect pigment stability, dispersion, color baseline, mechanical properties, and migration behavior. Therefore, pigment-based packaging should be tested under processing conditions that resemble industrial production rather than only under mild laboratory preparation. Large-scale manufacturing also requires stable pigment supply, batch-to-batch consistency, cost-effective extraction or purification, compatibility with existing packaging lines, and quality-control methods for monitoring pigment loading, release kinetics, and sensing performance. For intelligent indicators, sensing accuracy remains a major challenge. Colorimetric responses should be quantitatively correlated with reliable food-quality and safety indicators, such as pH, TVB-N, microbial counts, lipid oxidation, texture deterioration, and sensory rejection. Moreover, the robustness of RGB-based or image-based models should be verified under different lighting conditions, camera devices, packaging backgrounds, food types, humidity levels, and storage temperatures to avoid false-positive or false-negative freshness judgments.

Future research should shift from simply incorporating pigments into food-related materials toward mechanism-guided, matrix-specific, and application-oriented design. Curcumin-based systems may benefit from improved photostability, controlled photoactivation, optimized release, and stable surface retention at food interfaces. Anthocyanin-based indicators require better calibration between color changes and quality parameters, as well as greater attention to humidity tolerance, color reversibility, baseline drift, and model reproducibility. Carotenoid-based systems deserve further exploration in lipid-rich and oxidation-sensitive foods, particularly through nanoemulsions, microcapsules, DES-assisted delivery, and compatible biopolymer matrices. More rigorous control experiments are needed to clarify pigment–matrix interactions and synergistic mechanisms. At the same time, agricultural and seafood by-products can provide sustainable pigment sources, linking active food systems with circular bioeconomy.

Emerging digital technologies may further improve the reliability and practical value of pigment-based intelligent packaging, but their maturity levels differ. Among the technologies discussed in current pigment-based packaging studies, smartphone-assisted image analysis has already been experimentally demonstrated. For example, anthocyanin-based films for beef and pork freshness monitoring have used smartphone or RGB-based analysis to convert visual color changes into quantitative color parameters, thereby reducing the subjectivity of naked-eye judgment [5,76]. In these cases, digital analysis mainly serves as a quantitative readout tool for colorimetric indicators. By contrast, machine learning-assisted color interpretation, AI-assisted freshness prediction, and IoT-enabled intelligent packaging remain more prospective in the specific field of natural pigment-based food packaging. These approaches could potentially integrate color data with storage time, temperature, humidity, food type, and cold-chain information to support dynamic shelf-life prediction and real-time food-quality monitoring. However, their practical implementation still requires standardized image acquisition, robust calibration datasets, validation across diverse food matrices and storage conditions, sensor integration, data security, consumer-friendly interfaces, and regulatory acceptance. Therefore, future research should distinguish between experimentally validated digital readout methods and broader AI/IoT-based monitoring concepts that still require systematic validation before commercial application. Overall, future opportunities lie in developing safe, stable, scalable, digitally compatible, and quantitatively reliable pigment-based systems tailored to specific food deterioration pathways.

## 7. Conclusions

This review summarizes recent progress in curcumin-, anthocyanin-, and carotenoid-based active and intelligent packaging systems for food preservation and freshness monitoring. Rather than treating these pigments as universal multifunctional additives, this review emphasizes that their practical effectiveness depends on how pigment chemistry is translated into suitable material architectures and matched with food-matrix-specific deterioration pathways. Curcumin-based systems are generally more relevant to antioxidant, antimicrobial, and photoresponsive preservation; anthocyanin-based systems are more closely associated with pH- and amine-responsive freshness monitoring; and carotenoid-based systems show emerging potential for antioxidant-oriented protection. However, in all cases, the final performance is determined not by the pigment alone but by the interaction among pigment stability, carrier design, matrix compatibility, release behavior, and the specific spoilage mechanisms of the target food.

Future research should therefore strengthen the material-to-matrix bridge by optimizing pigment loading, dispersion, controlled release, photostability, color stability, and migration control under real food-storage conditions. More quantitative and standardized evaluations are also needed, including antimicrobial efficiency, antioxidant retention, sensing accuracy, shelf-life extension, sensory acceptance, and safety-related migration data. For industrial translation, pigment-based packaging systems should be tested under pilot-scale processing, storage, transportation, and commercial packaging conditions, rather than only in simplified laboratory models. Regulatory and safety issues, including carrier toxicity, nanoparticle migration, degradation products, labeling requirements, and compliance with food-contact material regulations, should be addressed at an early stage of material design. In addition, sustainability assessment should be strengthened through biodegradability testing, recyclability analysis, and life-cycle assessment of both pigments and carrier materials. Interdisciplinary collaboration among food science, materials engineering, microbiology, toxicology, sensory science, and regulatory science will be essential for developing safe, scalable, and consumer-acceptable systems. Overall, future development should move beyond simple pigment incorporation and establish clearer relationships among pigment chemistry, material engineering, food-matrix requirements, and deterioration pathways to guide realistic, safe, and application-oriented packaging systems.

## Figures and Tables

**Table 1:** Comparative summary of representative routes and reported performance of curcumin-, anthocyanin-, and carotenoid-based food systems.

Pigments	Preservation Routes	Preservation Effect	Ref.
Curcumin	Antimicrobial packaging	Extends banana shelf life by 10 days	[16]
	Antimicrobial coating	Applied to bananas, with inhibition zones of 9.45–13.67 mm; weight loss reduced by 16.42%/17.58% after 7 days	[17]
	Antimicrobial and antioxidant	Applied to apples/cherries, with antioxidant activity of 91.03% and inhibition zones of 7.8–9.1 mm	[18]
	Antimicrobial packaging	Applied to strawberries, with 100% inhibition rate against E. coli; shelf life of 8 days	[9]
	Antimicrobial and antioxidant	Applied to blueberries, with inhibition zones of 19.30/20.53 mm; weight loss rate of 30.6% after 18 days	[24]
	Antimicrobial nanofiber film	Applied to grapes, with bacteriostatic rates of 91.93%/93.00%; good fruit rate of 60.25% after 12 days; shelf life extended by 4 days	[27]
	Antimicrobial	Extends pork shelf life to 10 days; color change ΔE > 5	[21]
		Survival rates of pork spoilage bacteria all <45%; shelf life of 12 days	[38]
	Antimicrobial photodynamic therapy	Antimicrobial rate against beef > 95%; free radical scavenging rate > 90%	[41]
		Bacteriostatic rate against salmon approximately 99.99%	[4]
		Bacteriostatic rate against salmon of 99.8%	[50]
		Extends yellow croaker shelf life by 2 days	[46]
		Fish shelf life of 9 days	[10]
	Antioxidant	Grass carp shelf life of 10–14 days	[51]
		Schizothorax fish fillet shelf life of 15 days	[52]
	Antimicrobial/indicator	Extends schizothorax fish fillet shelf life from 3–6 days to 12–15 days; color changes from yellow to orange to red	[53]
	Antimicrobial/antioxidant	Tilapia shelf life of 8 days	[56]
Anthocyanins	Antioxidant + indicator	Extends strawberry shelf life to 13 days; the film turns from purple to yellowish purple to indicate freshness loss	[2]
	Antioxidant	Extends strawberry shelf life by 3 days	[61]
	Antimicrobial	Shelf life of grapes/blueberries: 20 days/12 days	[62]
	Antimicrobial/intelligent indicator	Extends pork shelf life by 3 days; color changes from red to brown to brownish-yellow	[68]
	Antimicrobial	The shelf life of pork is extended to 6 days	[70]
		Pork shelf life of 2 days	[71]
		The shelf life of pork is extended by 4 days at 4 °C and 12 h at 20 °C	[72]
		The fresh-keeping period of pork can reach 40 h	[73]
		The shelf life of pork was extended by 5 days	[74]
	Antioxidant + antimicrobial	Shelf life of pork is extended by 100% at 25 °C	[75]
	Antimicrobial	Delays beef spoilage by 2–4 days; preservation time is nearly twice that of the control	[78]
	Antioxidant + antimicrobial	Extends shrimp shelf life by 100% at 4 °C	[88]
Anthocyanins	Antimicrobial	Extends shrimp shelf life by approximately 12 h compared with the control	[89]
	Intelligent label + antimicrobial	Extends salmon shelf life from 6 days to 12 days; the label turns from red to grayish-purple	[80]
	Antimicrobial	Extends channel catfish shelf life by 2 days	[81]
	Antimicrobial photodynamic therapy	The shelf life of fish meat is approximately 8 days	[82]
Astaxanthin	Antioxidant + bacteriostatic	Strawberries preserved at room temperature for approximately 7 days	[3]
		Extends strawberry shelf life from 12 days to 18 days	[95]
Lutein	Freshness monitoring	Surimi appears green after 3–5 days of cold storage, bright green after 7 days, and turns yellow after 9 days	[96]
Lycopene	Antimicrobial + antioxidant	Cherries preserved at 0 °C for 15 days	[15]

## Data Availability

No new data were created or analyzed in this study. Data sharing is not applicable to this article.
